# LRH1 Acts as an Oncogenic Driver in Human Osteosarcoma and Pan-Cancer

**DOI:** 10.3389/fcell.2021.643522

**Published:** 2021-03-15

**Authors:** Yang Song, Weiwei An, Hongmei Wang, Yuanren Gao, Jihua Han, Chenguang Hao, Lin Chen, Shilong Liu, Ying Xing

**Affiliations:** ^1^The First Department of Orthopedic Surgery, The Second Affiliated Hospital of Harbin Medical University, Harbin, China; ^2^Institute of Cancer Prevention and Treatment, Heilongjiang Academy of Medical Science, Harbin Medical University, Harbin, China; ^3^Department of Pathology, Harbin Medical University Cancer Hospital, Harbin, China; ^4^Department of Intervention, Harbin Medical University Cancer Hospital, Harbin, China; ^5^Department of Head and Neck Surgery, Harbin Medical University Cancer Hospital, Harbin, China; ^6^Department of Thoracic Radiation Oncology, Harbin Medical University Cancer Hospital, Harbin, China; ^7^The Fourth Department of Medical Oncology, Harbin Medical University Cancer Hospital, Harbin, China

**Keywords:** LRH1, osteosarcoma, metastasis, epithelial-mesenchymal transition, angiogenesis, pan-cancer

## Abstract

Osteosarcoma (OS) that mainly occurs during childhood and adolescence is a devastating disease with poor prognosis presented by extreme metastases. Recent studies have revealed that liver receptor homolog 1 (LRH-1) plays a vital role in the metastasis of several human cancers, but its role is unknown in the metastasis of OS. In this study, Gene Ontology (GO) enrichment analyses based on high-throughput RNA-seq data revealed that LRH-1 acted a pivotal part in the positive regulation of cell migration, motility, and angiogenesis. Consistently, LRH-1 knockdown inhibited the migration of human OS cells, which was concurrent with the downregulation of mesenchymal markers and the upregulation of epithelial markers. In addition, short hairpin RNAs (shRNAs) targeting LRH-1 inactivated transforming growth factor beta (TGF-β) signaling pathway. LRH-1 knockdown inhibited human umbilical vein endothelial cell (HUVEC) proliferation, migration, and tube formation. Vascular endothelial growth factor A (VEGFA) expression was also downregulated after LRH-1 knockdown. Immunohistochemistry (IHC) revealed that the expression of LRH-1 protein was significantly higher in tumor tissues than in normal bone tissues. We found that high LRH-1 expression was associated with poor differentiation and advanced TNM stage in OS patients using IHC. Based on The Cancer Genome Atlas (TCGA) database, high LRH-1 expression predicts poor survival in lung squamous cell carcinoma (LUSC), kidney renal papillary cell carcinoma (KIRP), and pancreatic adenocarcinoma (PAAD). The downregulation of LRH-1 significantly hindered the migration and motility of LUSC cells. Using multi-omic bioinformatics, the positive correlation between LRH-1- and EMT-related genes was found across these three cancer types. GO analysis indicated that LRH-1 played a vital role in “blood vessel morphogenesis” or “vasculogenesis” in KIRP. Our results indicated that LRH-1 plays a tumor-promoting role in human OS, could predict the early metastatic potential, and may serve as a potential target for cancer therapy.

## Introduction

Osteosarcoma (OS) is the most common malignant bone tumor and ranks the leading causes of cancer-related deaths in the pediatric age group (Geller and Gorlick, 2010; Jafari et al., 2020). OS features generally a high-grade malignancy and an extremely aggressive phenotype, with a high frequency of metastasis and invasion (Botter et al., 2014; Fan et al., 2020). Although many therapeutic strategies have shown immense progress and surgery can control local primary tumor, the metastasis of OS can lead to death in most cases (Lindsey et al., 2017). Metastatic OS has unfavorable response to the current standard chemotherapy, and its 5-year survival rate is about 20% (Geller and Gorlick, 2010). Thus, there is an urgent research aim to identify novel biomarkers and understand the molecular mechanisms of metastasis, and new therapeutic approaches are also urgently needed in OS.

Liver receptor homolog 1 (LRH-1) [also called Nuclear Receptor Subfamily 5 Group A Member 2 (NR5A2)] that is located on chromosome 1q32.11 is a member of the nuclear receptor (NR) subfamily (Galarneau et al., 1998; Zhang et al., 2001). This NR is pivotal for stem cell pluripotency, energy metabolism, embryonic development, reproduction, and adult homeostasis; deregulation of LRH-1 has been associated with inflammation and cancer (Gu et al., 2005; Heng et al., 2010; Nadolny and Dong, 2015). Previous studies have demonstrated high LRH-1 expression in various cancer cells and tissues and provided supporting evidence of the oncogenic roles of LRH-1 (Lin et al., 2014a; Nadolny and Dong, 2015; Liu et al., 2018; Wu et al., 2018). Uncontrolled LRH-1 activity, by effecting multiple distinct signaling pathways, drives proliferation and stemness of cancer cells (Lin et al., 2014b; Bianco et al., 2015; Luo et al., 2017; Sun et al., 2020). With a particular focus on metastasis, a number of scientific studies were able to provide evidence that an implication of LRH-1 overexpression enhances metastatic potential as well as invasion of cancer cells, contributing to a more aggressive malignant phenotype in different types of human cancer, including gastric cancer (Liu et al., 2019), pancreatic cancer (Lin et al., 2014a), ovarian cancer (Sun et al., 2020), lung cancer (Ye et al., 2019), and colon cancer (Yan et al., 2017). However, it is unclear whether LRH-1 contributes to OS metastasis and tumor aggressiveness.

Metastasis proceeds through multiple steps and involves a number of factors including the degradation of the extracellular matrix, the epithelial-to-mesenchymal transition (EMT), migration and invasion, tumor angiogenesis, metabolic reprogramming, immunosuppression, and so on (Su et al., 2015). EMT is regarded as the first step of the metastatic cascade and is closely associated with malignant tumor cell migration and invasion (Nieto et al., 2016; Pastushenko and Blanpain, 2019; Cui et al., 2020). Transforming growth factor beta (TGF-β) signaling pathway is found to play a predominant role in EMT and cancer cell metastasis (Hao et al., 2019). Previous studies have shown that angiogenesis as an essential component is required for invasive tumor metastasis (Folkman, 2002). The members of vascular endothelial growth factor (VEGF) families, particularly VEGFA, act as the most common tumor angiogenic factors (Folkman, 2002). However, to our knowledge, the function of LRH-1 in tumor angiogenesis is unknown.

This study was conducted to investigate the metastatic potential of LRH-1 in OS. We used cell-based assays and tissue specimens derived from OS patients to investigate the role and mechanism(s) by which LRH-1 promotes OS metastatic spread. Our data demonstrated the involvement of LRH-1 in the metastatic cascade, such as the acquisition of migrative properties and the induction and promotion of angiogenesis in OS. This indicates LRH-1 as a potential target for the therapeutic management of metastatic OS.

## Materials and Methods

### Cell Culture

The human OS cell lines 143B and SJSA-1 and human umbilical vein endothelial cells (HUVECs) were purchased from the American Type Culture Collection (ATCC, Manassas, VA, United States). All cell lines were cultured at 37°C with 5% CO_2_ incubator (Thermo Fisher Scientific). The SJSA-1 and U-2 OS cells were grown in Dulbecco’s modified Eagle medium (DMEM) containing 10% fetal bovine serum (FBS; Gibco) and 1% penicillin/streptomycin (P/S; Gibco). 143B cells were grown in RPMI 1640 medium (Gibco) supplemented with 10% FBS and 1% P/S (Gibco). SK-MES-1 cells were grown in Minimum Essential Medium (MEM) containing 10% FBS (Gibco) and 1% P/S (Gibco). HUVECs in Ham’s F-12 K supplemented with 100 μg/ml heparin (Sigma) were cultured under the same conditions. The short hairpin RNA (shRNA) plasmid targeting human LRH-1 used in lentivirus-mediated interference and control lentivirus was purchased from Genechem (www.genechem.com.cn; Shanghai, China). All plasmid vectors were validated by sequencing.

### Tissues and Immunohistochemistry (IHC)

Human formalin-fixed paraffin-embedded (FFPE) OS tissue microarray slides (OS804C and B024H) were purchased from Xi’an Taibosi Biotechnology Co., Ltd., with core diameters of 1.5 mm and thickness of 5 μm. OS804C contains 40 cases of OS tissue sample (2 cores/case), and clinicopathological characteristics of OS patients were shown in Table 1. B024H contains 11 cases of normal bone or ribs tissues (2 cores/case). The study was approved by the Ethics Committee of Harbin Medical University Cancer Hospital.

**TABLE 1:** Association between LRH1 expression and clinicopathological characteristics of OS patients.

		LRH1 expression		
		
Variables	No. of patients	Low	High	*P*-value
Gender				0.370
Female	14	6	8	
Male	26	15	11	
Age				0.698
<18 years	16	9	7	
≥18 years	24	12	12	
Location				0.330†
Femur	26	16	10	
Tibia	5	2	3	
Other sites	9	3	6	
Differentiation				0.005*
Well/Moderate (G1/2)	22	16	6	
Poor (G3)	18	5	13	
T stage				1^†^
T1	7	4	3	
T2	33	17	16	
TNM stage				0.01*^†^
I	22	16	6	
II	17	5	12	
III/IV	1	0	1	

*P < 0.05 was considered statistically significant.

*^†^Fisher’s exact test.*

The detailed experimental procedures of tissue dewaxing and antigen retrieval of the slides were described previously (Cheng et al., 2018; Xing et al., 2018). The slides were stained for LRH-1 immunohistochemistry (IHC) with the corresponding anti-LRH-1 (1:100 dilutions, ab189876; Abcam, Cambridge, United Kingdom). The levels of LRH-1 staining were scored as previously described (Liu et al., 2018).

### RNA-Seq Data Processing and Analysis

In this experiment, three biological replicates were performed for each group. Total RNA was prepared from samples, a preliminary assessment of library concentration and purity of the total RNA was determined with NanoDrop 2000, RNA integrity was detected by agarose gel electrophoresis, RIN value was assessed using the Agilent 2100 system, and then, small fragments of about 300 bp mRNA were copied into cDNA using fragmentation buffer, reverse transcriptase, and oligo dT magnetic beads. At the same time, a special adapter was added to the 3′ end of cDNA. Then, PCR was performed, targeted products were purified, and clustering of samples was performed on cBot Cluster Generation System according to PCR amplification. After cluster generation, library preparations were sequenced on the Illumina Novaseq 6000 platform with a 150-bp paired module (Majorbio Bio-pharm Technology Corporation). RNA sequencing (RNA-seq) data analysis was performed using the free online platform of Majorbio Cloud Platform^1^.

### Transwell Migration Assay

Transwell assays were performed to detect cell migration. The 24-well transwell chambers (8 μm pore size; Corning Costar, United States) were prepared with or without 100 μl matrigel matrix (BD Biosciences, San Jose, CA, United States) at 4°C overnight. After serum starvation for 6 h, 5 × 10^4^ cells in serum-free media were seeded in the upper chamber, whereas media with 10% FBS were placed in the lower chamber of each transwell. Transwell chambers were maintained at 37°C for 24 h in migration assay. Then, cells were fixed with methanol and stained with 0.1% crystal violet. The number of migrated cells was determined in five random fields under a microscope.

### Wound Healing Assay

A total of 2 × 10^5^ cells per well were seeded in a 6-well plate until grown to 80% confluency in complete media. After serum starvation overnight, a pipette tip was used to make a wound on the cell monolayer. Cells were washed with PBS twice, and media were added to the plates. The images of the wounds were captured at 0, 12, and 24 h post-scratch. ImageJ (v1.8.0) was used to quantify the wound area.

### Conditioned Media (CM) and Enzyme-Linked Immunosorbent Assay (ELISA)

Osteosarcoma cells were grown to 80% confluence in complete media and then incubated in serum-free media for 12 h prior to collection of media. Cell-free conditioned media (CM) were then collected, centrifuged at 2,000 rpm at 4°C for 10 min to remove cellular debris, and stored at −80°C. VEGFA quantification was analyzed by a human VEGFA ELISA kit (USCN Life Science Inc., Wuhan, China). The measurements were performed following the manufacturer’s protocol, and the optical density was measured at 450 nm with a plate reader (BioTek, Winooski, VT, United States).

### HUVEC Proliferation and Tube Formation Assay

Osteosarcoma conditioned media were used for HUVEC functional assays. HUVEC proliferation and tube formation assays were performed as previously described (Wang et al., 2019). Cell Counting Kit-8 (Dojindo Molecular Technologies, Kumamoto, Japan) was used to detect the proliferation of HUVEC. The photo of HUVEC migration and tube formation was captured using a light microscope (Olympus) and analyzed by ImageJ.

### Western Blot Analysis

Cells were harvested with radioimmunoprecipitation assay (RIPA) buffer (Beyotime Biotechnology, Shanghai, China) supplemented with protease inhibitors and pelleted by centrifugation at 4°C for 15 min. The protein concentrations were determined using a Pierce Bicinchoninic Acid Protein Assay (Thermo Fisher Scientific, Waltham, MA, United States). Proteins were separated by sodium dodecyl sulfate polyacrylamide gel electrophoresis (SDS-PAGE), followed by polyvinylidene fluoride (PVDF) membranes transfer, blocked in 5% bovine serum albumin (BSA), and then incubated with specific antibodies. Antibodies were used as follows: LRH-1 (ab153944; Abcam, Cambridge, MA, United States), E-cadherin (1:1,000, ab40772; Abcam), N-cadherin (1:1,000, ab18203; Abcam), Phospho-Smad2 (Ser465/467) (138D4; Cell Signaling Technology), and β-actin (TA-09; ZSGB-Bio, China).

### qRT-PCR

The quantitative real-time PCR (qRT-PCR) analysis was performed as previously described (Liu et al., 2018). Total RNA was isolated from OS cells by using the Total RNA kit I (Omega Bio-tek Inc., Hilden, Germany) and reverse transcribed to cDNA with Transcriptor First Strand cDNA Synthesis Kit (Roche Diagnostics, Penzberg, Germany). qRT-PCR was analyzed using FastStart Universal SYBR^®^ Green Master (Roche). All samples were normalized to GAPDH, and the 2^–ΔΔ*Ct*^ method was used to evaluate the relative levels of genes.

### Multi-Omic Bioinformatics Analysis

The transcriptome profiling data and clinical information of pan-cancer patients were extracted from The Cancer Genome Atlas (TCGA) database. The Human Protein Atlas (HPA)^2^ was used to confirm prognostic significance and protein expression levels. The LinkFinder module of the LinkedOmics database^3^ was used to explore genes differentially expressed to be correlated with LRH-1 in the TCGA KIRP cohort (*n* = 290), LUSC cohort (*n* = 501), and PAAD cohort (*n* = 178) using Pearson’s correlation coefficient. The results were graphically presented in volcano plots and heat maps. Correlation analysis has been performed by using R software, and corrplot packages^4^ were exploited to figure out the data. Pearson correlation was used to compare the relationship between LRH-1 and genes related to the EMT phenotype.

ClusterProfiler package (Yu et al., 2012) performed Kyoto Encyclopedia of Genes and Genomes (KEGG) pathway analysis for the related genes. KEGG pathway analysis revealed biological pathways associated with related genes. Adjusted *P*-value < 0.05 and the absolute value of Pearson correlation coefficient > 0.4 were used as the cutoff standards. The data source is TCGA database. Metascape^5^ is an easy-to-operate online web tool that can be used for gene annotation and analysis to help biologists understand one or more gene lists (Zhou et al., 2019). Metascape provides automated meta-analysis tools to understand a set of common and unique approaches in orthogonal target discovery research. In this study, we used it to perform enrichment analysis of LRH-1 and its related genes. With default setting, MEXPRESS^6^ was conducted to explore the correlation of DNA methylation and LRH-1 expression (Koch et al., 2015).

### Statistical Analysis

The experimental results were presented as mean ± SEM. All statistical analyses were completed by SPSS v26.0 (IBM Corp. Armonk, NY, United States) and GraphPad Prism software v8.0.2 (GraphPad Software, San Diego, CA, United States). Survival curves were plotted by the Kaplan–Meier method, and survival analysis was performed with log-rank test. Significant differences among categorical variables and continuous variables were analyzed by Student’s *t*-test and chi-square test. A two-sided *P*-value < 0.05 was considered to be statistically significant.

## Results

### Elevated LRH1 Expression Is Associated With Advanced TNM Stage in OS Patients

**FIGURE 1:**
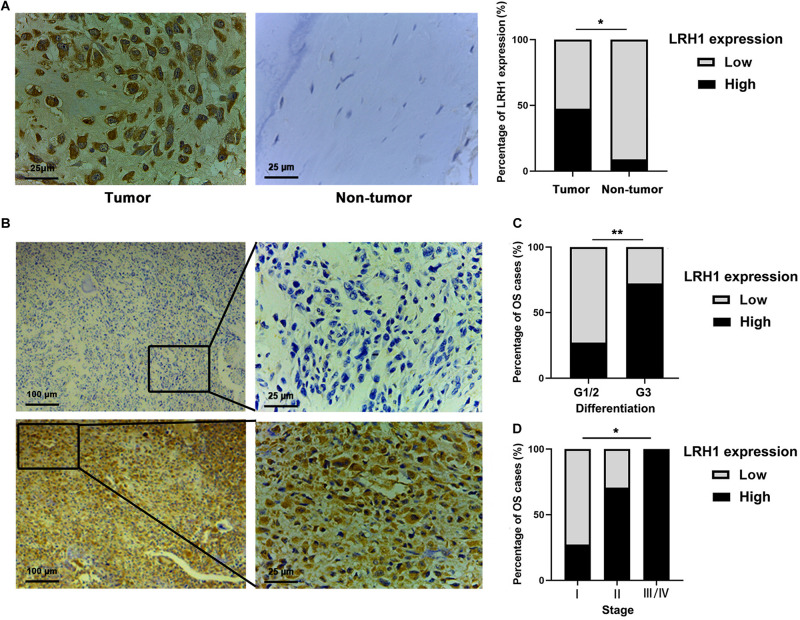
Elevated LRH1 expression correlates with advanced TNM stage of osteosarcoma (OS) patients. **(A)** Representative immunohistochemistry (IHC) images showing the expression of LRH1 in OS and nontumoural bone tissues (non-tumour). Original magnification, ×400; Scale bar = 25 μm. A histogram shows the percentage of LRH1 expression in tumour and nontumour tissues. **(B)** Representative IHC staining images are shown of high expression (left) and low expression (right) of LRH1 in OS tissues. Original magnification, ×100; Scale bar = 100 μm. **(C,D)** The percentages of patients with different TNM stages **(C)** and different differentiation **(D)** were assigned according to the expression level of LRH1 in OS. *P < 0.05, **P < 0.01.

LRH-1 protein expression in OS tissue and normal bone or ribs tissue samples was tested using IHC analysis. Our results revealed that LRH-1 was clearly localized to the nuclear and cytoplasmic compartment of OS cells (Figures 1A,B). LRH-1 was highly expressed in 47.5% of OS cases (19/40). Compared with those in non-tumor tissues, LRH-1 expression was elevated in OS tissues (Figure 1A). Next, we examined the association between LRH-1 expression and clinicopathological characteristics of OS patients. We found that patients with high LRH-1 expression had more advanced TNM stage and poor differentiation than patients with low LRH-1 expression (Table 1 and Figure 1B). These significant correlations suggested that LRH-1 might be a potential clinical biomarker.

### RNA-Seq Analysis Identifies LRH1 as a Metastatic-Promoting Regulator in OS Cells

**FIGURE 2:**
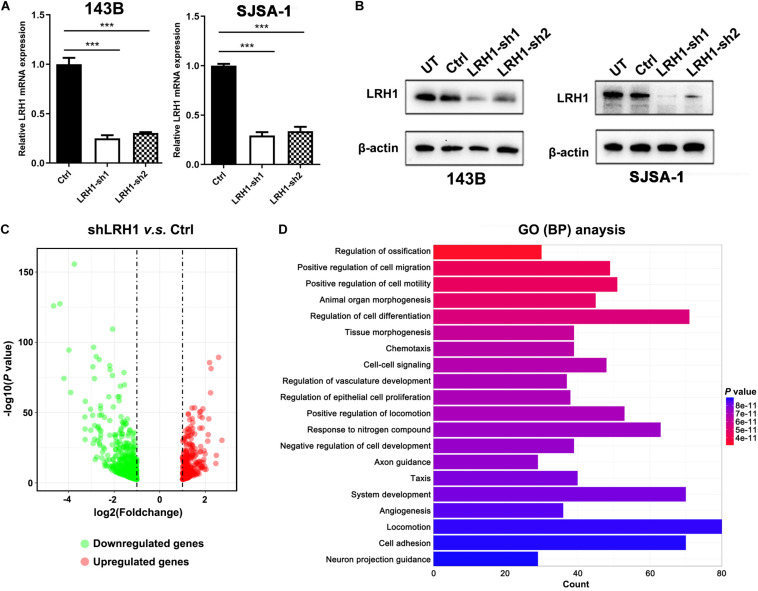
LRH1 is a metastatic-promoting regulator in OS cells. **(A)** RT-PCR and **(B)** western blot analysis results are shown of LRH1 in 143B and SJSA-1 cells untreated (UT) or transfected with control shRNA (Ctrl) or two shRNAs (LRH1-sh1 and LRH1-sh2) against LRH1. Mean ± SD, *n = 3*, ***P < 0.001. **(C)** A volcano plot indicates upregulated (red dots) and downregulated (gray dots) genes in the shLRH1 group compared with the control group. Black dotted lines represent a cut-off range of 1.0-fold and *P* < 0.05. **(D)** The top 20 enriched pathways identified by GO (BP) terms based on differentially expressed genes are shown.

To explore the role of LRH-1 in OS cells, shRNAs targeting LRH-1 were transfected into 143B and SJSA cells. Using qRT-PCR and Western blot, we found that shRNA1 and shRNA2 both efficiently knocked down LRH-1 mRNA (Figure 2A) and protein expression (Figure 2B), and shRNA1 (shLRH-1) was applied in the subsequent RNA sequencing experiment. We conducted global gene expression profiling in OS cells infected with a lentivirus expressing either scrambled shRNA (Ctrl) or shLRH-1 using RNA-seq transcriptome analysis. We identified 789 differentially expressed genes [adjusted *P* < 0.05 and absolute log_2_ (fold change) > 1], which involved 262 upregulated genes and 527 downregulated genes after LRH-1 inhibition (Figure 2C). Based on these differentially expressed genes, Gene Ontology (GO) terms affected by LRH-1 were shown in Figure 2D and Supplementary Figure 1. Three categories of GO annotation were biological process (BP), cellular component (CC), and molecular function (MF) categories (Li et al., 2020). GO terms were ranked according to *P*-value. In the BP category, metastasis-related events including “positive regulation of cell migration,” “positive regulation of cell motility,” “angiogenesis,” and so on appear in the top 20 terms (Figure 2D). The results also revealed that metastasis-related CC and MF terms (i.e., extracellular matrix) significantly ranked the top 20 terms (Supplementary Figure 1). These findings indicate that OS metastasis might be influenced by LRH-1.

### LRH1 Depletion Decreases the Motility and Migratory Properties of OS Cells

**FIGURE 3:**
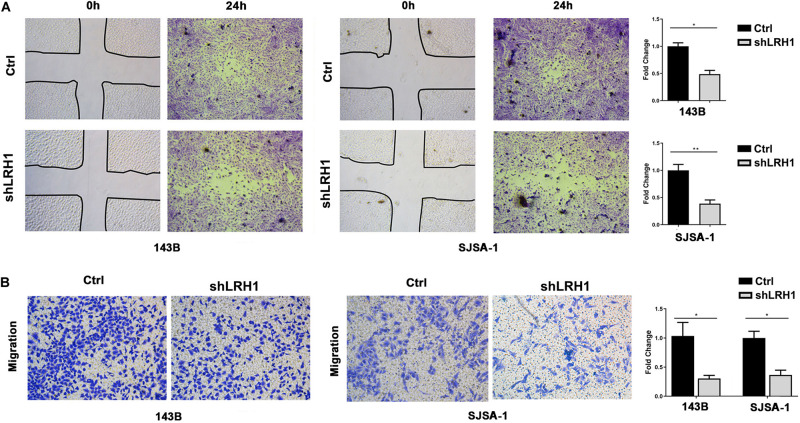
LRH1 depletion suppresses the motility and migration of OS cells. **(A)** Wound healing assays indicate the motility and migration of OS cells in shLRH1 group and control group. **(B)** Transwell assays revealed the migration of Ctrl and shLRH1 groups. Representative images from 3 experiments are shown in **(A)** and **(B)**. Migrated cell numbers are quantified in **(A)** and **(B)**, respectively (mean ± SEM, *n = 3*). *P < 0.05 and **P < 0.01 versus Ctrl, by 2-way ANOVA with Tukey’s *t* test.

Next, we tested whether LRH-1 affects OS cell motility and migration. As expected, wound healing assays revealed that knocking down LRH-1 suppressed the motility and migration of OS cells (Figure 3A). When compared with control cells, the migratory ability of 143B and SJSA-1 cells was detected by transwell assay (Figure 3B). These results suggest that LRH-1 promotes the metastatic ability of OS cells.

### LRH1 Loss Suppresses the EMT and the TGF-β Signalling Pathway

**FIGURE 4:**
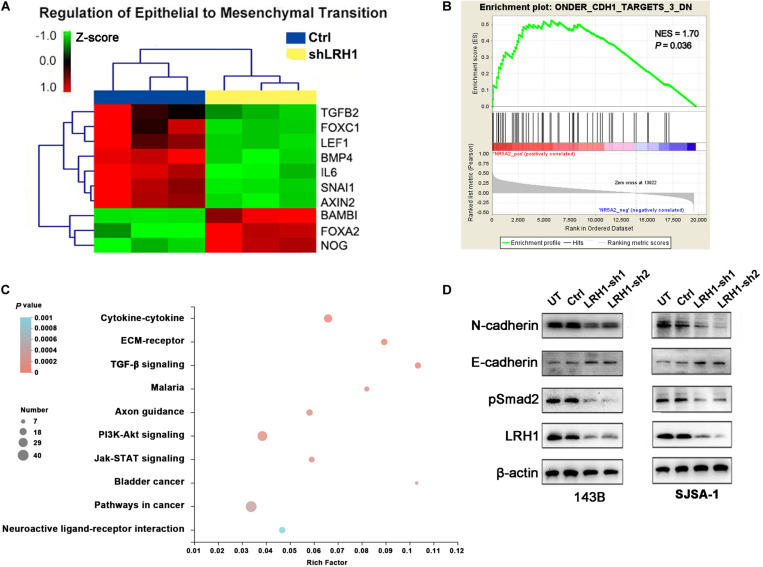
Effect of LRH1 loss on EMT and the TGFβ signalling pathway. **(A)** Heatmaps of GO (BP) categories show the differential expression of epithelial to mesenchymal transition pathway gene signatures in control and shLRH1 OS cells. Red and green indicate high and low mRNA expression levels, respectively. **(B)** The GSEA results show a correlation between LRH1 levels and the gene set “ONDER CDH1 TARGETS 3 DN.” **(C)** The top 10 enriched pathways identified by KEGG (Kyoto Encyclopedia of Genes and Genomes) pathway analysis based on differentially expressed genes. Rich factor = number of enriched genes/number of background genes in the pathway. **(D)** western blot analysis of E-cadherin, N-cadherin and TGFβ signalling pathway was conducted in Ctrl OS cells and shLRH1 OS cells. β-actin served as a loading control.

Epithelial-to-mesenchymal transition is a cellular program that contributes to enhance cell migratory and invasive abilities. In the GO analysis results based on RNA-seq data, “regulation of epithelial-to-mesenchymal transition” was found in the BP category (Figure 4A). Based on the OS datasets downloaded from Therapeutically Applicable Research to Generate Effective Treatments (TARGET), the Gene Set “ONDER CDH1 TARGETS 3 DN,” which involves genes downregulated in cancer cells after loss of function of E-cadherin, was positively correlated with LRH-1 expression using Gene Set Enrichment Analysis (GSEA) (Figure 4B). Next, we performed KEGG pathway of differential gene expression. The top 10 pathways were visualized as bubble chart in Figure 4C. TGF-β signaling pathway ranked first according to rich factor value (Figure 4C). In OS cells, E-cadherin as one of the most important epithelial markers was upregulated, whereas mesenchymal markers, such as N-cadherin, were downregulated after LRH-1 knockdown compared with control cancer cells (Figure 4D). Consistently, depletion of LRH-1 suppressed the activity of TGF-β signaling pathway by Western blot (Figure 4D). Our results indicated that LRH-1 positively regulates metastasis, EMT, as well as TGF-β signaling pathway in OS.

### Decreased LRH1 Expression Inhibits Angiogenesis and VEGFA Production

**FIGURE 5:**
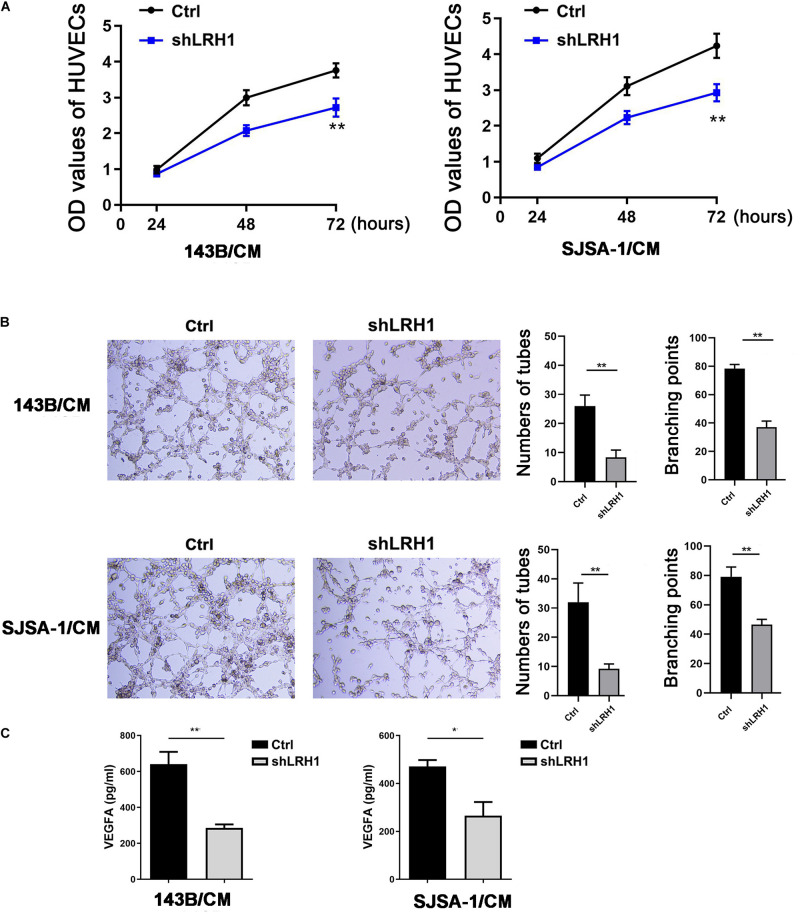
Decreased LRH1 inhibits HUVEC proliferation and tube formation. **(A,B)** HUVECs were treated with CM from OS cells transfected with shLRH1 or Ctrl. **(A)** A CCK-8 kit was used to evaluate the proliferation ability of HUVECs. **(B)** Tube formation tests were performed. Tube length and branch points were analyzed to evaluate angiogenic activity. **(C)** The expression of VEGFA in OS cells transfected with shLRH1 or Ctrl was determined by ELISA. The values are represented as the means of three independent experiments. *P < 0.05 and **P < 0.01 versus Ctrl.

As shown in Figure 2D, GO BP analysis indicated that LRH-1 played a role in angiogenesis. To validate the effect of LRH-1 on angiogenesis *in vitro*, HUVECs were treated with CM from OS cells transfected with shLRH-1 or Ctrl. The proliferation, migration, and tube formation abilities of HUVEC were considered as the indicators of *in vitro* angiogenesis activity in previous studies (Cai et al., 2017; Wang et al., 2019). Compared with the CM from Ctrl, the CM from shLRH-1 showed the decreased capability of HUVEC proliferation (Figure 5A) and tube formation (Figure 5B). The VEGFA is a vital pro-angiogenesis factor in OS (Wang et al., 2015). Using enzyme-linked immunosorbent assay (ELISA) assay, we revealed that the production of VEGFA was reduced after LRH-1 knockdown in OS cells (Figure 5C). These results highlight the significance of LRH-1 in potentiating the angiogenesis of OS.

### A Pan-Cancer Analysis of the Oncogenic Role of LRH1

**FIGURE 6:**
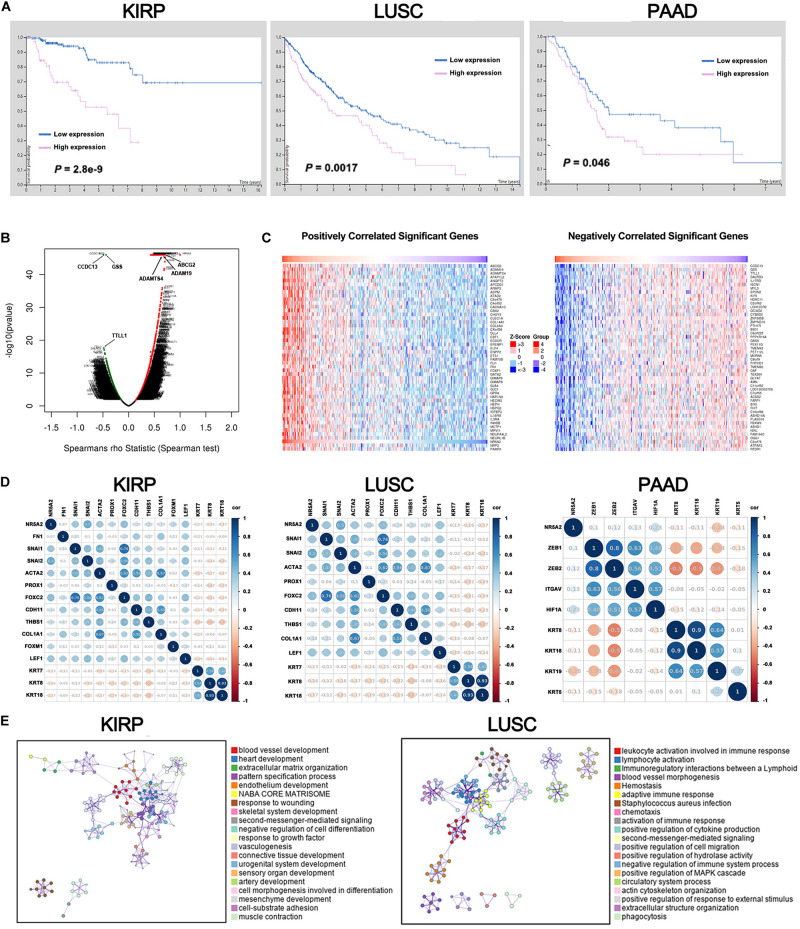
A pan-cancer analysis of the oncogenic role of LRH1. **(A)** Kaplan–Meier curves show the overall survival for KIRP, LUSC and PAAD patients with high or low LRH1 expression from the TCGA database. **(B)** A volcano plot shows genes (red dots) that positively correlate with LRH1 and genes (green dots) that positively correlate with LRH1 in KIRP. **(C)** Heat maps show genes positively and negatively correlated with LRH1 mRNA expression in KIRP (top 50). **(D)** A correlation map displays Pearson correlation values for each pair of genes in three types of cancer. The bar on the left of the map indicates the color legend of the Pearson correlationvalues calculated for each couple of genes. **(E)** An interactive network of the top 20 enriched terms is colored by cluster ID in KIRP and LUSC. Each color represents one enrichment pathway.

Liver receptor homolog 1 deregulation is often associated with a variety of cancers. Therefore, we conducted multi-omic bioinformatics analysis from public databases across pan-cancer for the first time. The online web tool HPA was our preferred option, because HPA provides information on 24,000 human proteins and their expression in 20 kinds of human tumor tissues. As shown in Supplementary Figure 2, most cancer tissues showed strong LRH-1 immunoreactivity (e.g., 11/11 pancreatic cancer patients show high/medium expression). We next applied the HPA approach to identify the prognostic values of LRH-1 across various cancer types based on TCGA. The result revealed that patients with high LRH-1 expression had worse overall survival (OS) than those with low LRH-1 expression in kidney renal papillary cell carcinoma (KIRP), lung squamous cell carcinoma (LUSC), and pancreatic adenocarcinoma (PAAD) (Figure 6A).

Liver receptor homolog 1 as an adverse prognostic factor suggested its tumor-promoting roles in KIRP, LUSC, and PAAD. Next, we demonstrated the BP of LRH-1 in these three kinds of tumors. Next, the function module of LinkedOmics was used to analyze RNA-seq data from 290 KIRP patients, 501 LUSC patients, and 178 PAAD patients in TCGA. The volcano plot showed that genes were significantly correlated with LRH-1 in KIRP, LUSC, and PAAD (Figure 6B and Supplementary Figure 3). The 50 significant gene sets were positively and negatively correlated with LRH-1 as shown in the heat map (Figure 6C and Supplementary Figure 3). This result suggests a widespread impact of LRH-1 on the transcriptome.

The main features of EMT are reduction in the expression of cell adhesion molecules (such as E-cadherin), cytoskeletal transformation from keratin to vimentin, and morphological characteristics of mesenchymal cells. KRT family genes are epithelial gene biomarkers in the EMT process. To measure the correlation between LRH-1- and EMT-related genes in KIRP, LUSC, and PAAD, we performed Pearson correlation metric (Figure 6D). Among these three cancer types, we found that LRH-1 was positively correlated with genes able to upregulate EMT phenotype reported in previous literature and negatively correlated with epithelial genes, denoting that LRH-1 may act as a mesenchymal gene to promote the EMT process in cancer.

In order to further explore the enrichment function of LRH-1-related genes, we performed GO and KEGG pathway analyses using the Metascape web-based tool. The networks of enrichment terms of LRH-1 according to cluster ID were displayed in Figure 6E. Notably, these results indicated that LRH-1 may play important roles through mediating blood vessel development in KIRC and LUSC.

**FIGURE 7:**
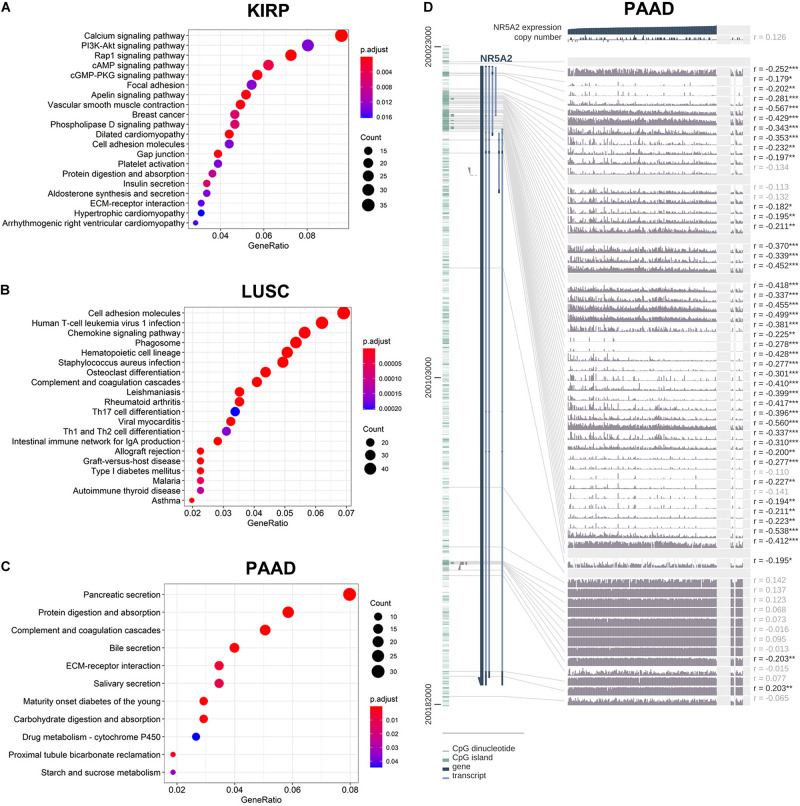
KEGG pathway analysis of related genes and DNA methylation of the LRH1 promoter. **(A–C)** KEGG pathway enrichment analysis of the DEGs in KIRP **(A)**, LUSC **(B)** and PAAD **(C)** was performed. **(D)** The correlation of LRH1 expression and DNA methylation of theLRH1 promoter region from MEXPRESS, including 223 samples in pancreatic adenocarcinoma from TCGA datasets is shown. The statistics [correlation coefficient (r) and *P*-value] on the right show the relationship between expression and DNA methylation of the promoter. *P < 0.05, **P < 0.01, and ***P < 0.001.

In order to explore the enrichment of the functions and pathways of the related genes of LRH-1, KEGG pathway analysis was performed. In KIRP, the pathway enrichment analysis showed that related genes were associated with different pathways, such as calcium signaling pathway, PI3K-Akt signaling pathway, Rap1 signaling pathway, cAMP signaling pathway, and cGMP-PKG signaling pathway (Figure 7A). In LUSC and PAAD, the pathway enrichment analysis was shown in Figures 7B,C.

The LRH-1-related genes in PAAD were significantly correlated with renal system development, etc (Supplementary Figures 4A,B). The networks of enrichment terms of LRH-1 according to cluster ID were displayed in Supplementary Figure 4B. In summary, these results indicated that LRH-1 may play pivotal roles in pancreatic cancer through the regulation of endocrine and growth factors.

We hypothesized that DNA methylation might contribute to LRH-1 deregulation. To explore the relationships between DNA methylation of the LRH-1 promoter and LRH-1 expression, we performed the integration and visualization of LRH-1 expression and DNA methylation data using MEXPRESS (Koch et al., 2015). In PAAD, we found 43 CpG islands of LRH-1 (i.e., cg20406878) that were negatively and significantly associated with LRH-1 gene expression (Figure 7D). Furthermore, 17 and 6 CpG islands of LRH-1 promoter locus hypermethylation were inversely correlated with LRH-1 expression in lung adenocarcinoma (LUAD) (Supplementary Figure 5) and KIPR (Supplementary Figure 6).

## Discussion

In the present study, LRH-1 was found to be mainly located in the nucleus of OS cells by IHC. LRH-1 was reported in previous studies to constantly exhibit nuclear localization and govern vital transcriptional programs of several types of malignancy, especially breast cancer (Bianco et al., 2014; Wang et al., 2018) and pancreatic cancer (Lin et al., 2014a). The LRH-1 protein harbors highly conserved stretch of 26 amino acids, a Drosophila fushi tarazu factor 1 (Ftz-F1) box, with high affinity for specific response elements with the Ftz-F1-consensus binding sequence [(T/C)CAAGG(T/C)C(A/G)] (Fayard et al., 2004; Xu et al., 2004). It is interesting to identify the genes that are transcriptionally activated by LRH-1 in OS as a future study.

Here, we revealed that LRH-1 expression was significantly elevated in tumor tissues from OS patients. To the best of our knowledge, LRH-1 was firstly found to be associated with poor differentiation and advanced TNM stage in OS patients. In agreement with our findings, LRH-1 was overexpressed in human gastric, pancreatic, and lung tumor tissues compared with normal controls (Wang et al., 2008; Luo et al., 2017; Liu et al., 2018). Our previous study also elucidated that high LRH-1 expression was notably correlated with poor differentiation, advanced TNM stage, and positive lymph node metastasis in lung cancer (Liu et al., 2018). In addition, LRH-1 expression was significantly related to FIGO stage, lymph node metastasis, and intraperitoneal metastasis in ovarian cancer (Sun et al., 2020). Our results and previous reports suggested that LRH-1 might be a potential clinical biomarker.

Metastases especially to the lungs remain the leading cause for OS-associated mortality; a better understanding of the molecular mechanisms of the metastasis of OS will help to provide the novel strategies for combating metastatic progression (Fan et al., 2020). Our results firstly provide evidence that LRH-1 promotes OS metastasis. There is growing evidence that indicates that LRH-1 governs the metastasis of multiple tumors (Nadolny and Dong, 2015). The well-characterized functions of LRH-1 in pancreatic cancer metastasis were described (Lin et al., 2014a). LRH-1 spurs pancreatic cancer metastasis by enhancing the transcriptional activity of β-catenin and upregulates downstream targets (c-Myc, MMP2/9) (Lin et al., 2014a). Metastasis of gastric cancer was also promoted by LRH-1–β-catenin axis (Liu et al., 2019).

In this study, we investigated that LRH-1 might efficiently facilitate tumor metastasis by inducing EMT by TGF-β signaling pathway. In agreement with our results, LRH-1 had an effect on promoting the EMT progress of cancer cells (Lazarus et al., 2014; Luo et al., 2017; Liu et al., 2019; Sun et al., 2020). LRH-1 knockdown led to the increased expression of epithelial markers E-cadherin and β-catenin and decreased expression of mesenchymal marker Vimentin (Luo et al., 2017). In ovarian cancer and gastric cancer, the findings that LRH-1 has the capacity to induce EMT were reported (Liu et al., 2019; Sun et al., 2020). In line with our finding, LRH-1-overexpression spurred EMT and activated TGF-β signaling in breast cancer cells (Lazarus et al., 2014). Our results could help to improve therapeutic intervention, and LRH-1/TGF-β signaling pathway/EMT could serve as biomarkers in guiding the clinical intervention of metastatic OS patients.

Our work uncovers a hitherto unappreciated role of LRH-1 in angiogenesis. Our GO analysis revealed that LRH-1 played a vital role in “blood vessel morphogenesis” and “vasculogenesis,” indicating that LRH-1 might be involved in the angiogenesis of LUAD and KIRP. Moreover, we revealed that LRH-1 promoted VEGFA expression. Our results will help improve strategies for the selection of OS patients who may particularly benefit from agents that selectively target the VEGFA pathway. Given the impact of angiogenesis in tumor growth (Folkman, 2002), we will explore whether angiogenesis is required for LRH-1-induced cancer growth.

Collectively, the critical role of LRH-1 in OS cells was described in this study. Our findings demonstrated the function of LRH-1 in inducing EMT and activating TGF-β signaling pathway. This study provided substantial evidence that LRH-1 potentiates OS angiogenesis and VEGFA production. These observations improve our understanding of how LRH-1 impacts cancer progression. We consider that further research on LRH-1 might guide a personalized approach to human metastatic OS and other cancers in the future.

## Data Availability Statement

The TCGA dataset was obtained from the GDC API. The names of the repository and accession number(s) can be found below at the NCBI Sequence Read Archive (SRA), SRR13529483–SRR13529488 (Experiment SRX9937803–SRX9937808).

## Ethics Statement

The studies involving human participants were reviewed and approved by the Ethics Committee of Harbin Medical University Cancer Hospital. The patients/participants provided their written informed consent to participate in this study. Written informed consent was obtained from the individual(s) for the publication of any potentially identifiable images or data included in this article.

## Author Contributions

YS, YX, and SL designed the study, analyzed the results, and were major contributors in writing and revising the manuscript. WA and HW provided technical support. YS, YX, YG, and JH performed the experiments. SL, CH, and LC analyzed the data. YX assisted with manuscript review and revision. All authors read and approved the final manuscript.

## Conflict of Interest

The authors declare that the research was conducted in the absence of any commercial or financial relationships that could be construed as a potential conflict of interest.

## Footnotes

1 http://www.majorbio.com

2 https://www.proteinatlas.org/

3 http://www.linkedomics.org/login.php

4 https://github.com/taiyun/corrplot

5 http://metascape.org

6 http://mexpress.be

## Supplementary Material

Supplementary Figure 1 Cellular component and molecular function of differentially expressed genes. The top 20 enriched cellular component terms and molecular function terms identified by GO (CC) and GO (MF) are shown based on differentially expressed genes.

Supplementary Figure 2 Most cancer cells from the HPA showed strong nuclear immunoreactivity.

Supplementary Figure 3 Significant genes related to LRH1. **(A)** A Pearson test was used to analyze correlations between LRH1 and genes differentially expressed in LUSC (upper panel) and PAAD (lower panel). Heat maps of **(B)** and **(C)** show genes positively and negatively correlated with LRH1 in LUSC and PAAD, respectively (top 50). Red indicates positively correlated genes, and green indicates negatively correlated genes.

Supplementary Figure 4 Functional enrichment analysis of LRH1-related genes in pancreatic cancer. **(A)** A heatmap is shown of enriched terms regarding Gene Ontology across LRH1 and its related genes constructed by Metascape. **(B)** An interactive network shows the top 20 enriched terms colored by cluster ID. Each color represents one enrichment pathway.

Supplementary Figure 5 LRH1 depletion suppresses the motility and migration of LUSC cells. **(A)** RT-PCR and **(B)** Western blot analysis results are shown of LRH1 in SK-MES cells untreated (UT) or transfected with control shRNA (Ctrl) or two shRNAs (LRH1-sh1 and LRH1-sh2) against LRH1. Mean ± SD, *n = 3*, **P* < 0.05. **(C)** Wound healing assays indicate the motility and migration of LUSC cells in the shLRH1 group and control group. ***P* < 0.01 versus Ctrl, by 2-way ANOVA with Tukey’s *t* test.

Supplementary Figure 6 The correlation of LRH1 expression and DNA methylation of the LRH1 promoter region including 758 samples in LUAD from TCGA datasets. The statistics [correlation coefficient (r) and *P*-value] on the right show the relationship between expression and DNA methylation of the promoter (*<0.05, **<0.01, and ***<0.001).

Supplementary Figure 7 The correlation of LRH1 expression and DNA methylation of the LRH1 promoter region including 380 samples in KIRP from TCGA datasets. The statistics [correlation coefficient (r) and *P*-value] on the right show the relationship between expression and DNA methylation of the promoter (*<0.05, **<0.01, and ***<0.001).

Supplementary Table 1 The association between LRH1 expression and clinicopathological characteristics of OS patients.
