# Supplementary figures and images for: LRH1 Acts as an Oncogenic Driver in Human Osteosarcoma and Pan-Cancer
Source: Front Cell Dev Biol. 2021 Mar 15;9:643522. doi: 10.3389/fcell.2021.643522 (PMC8005613; doi:10.3389/fcell.2021.643522)

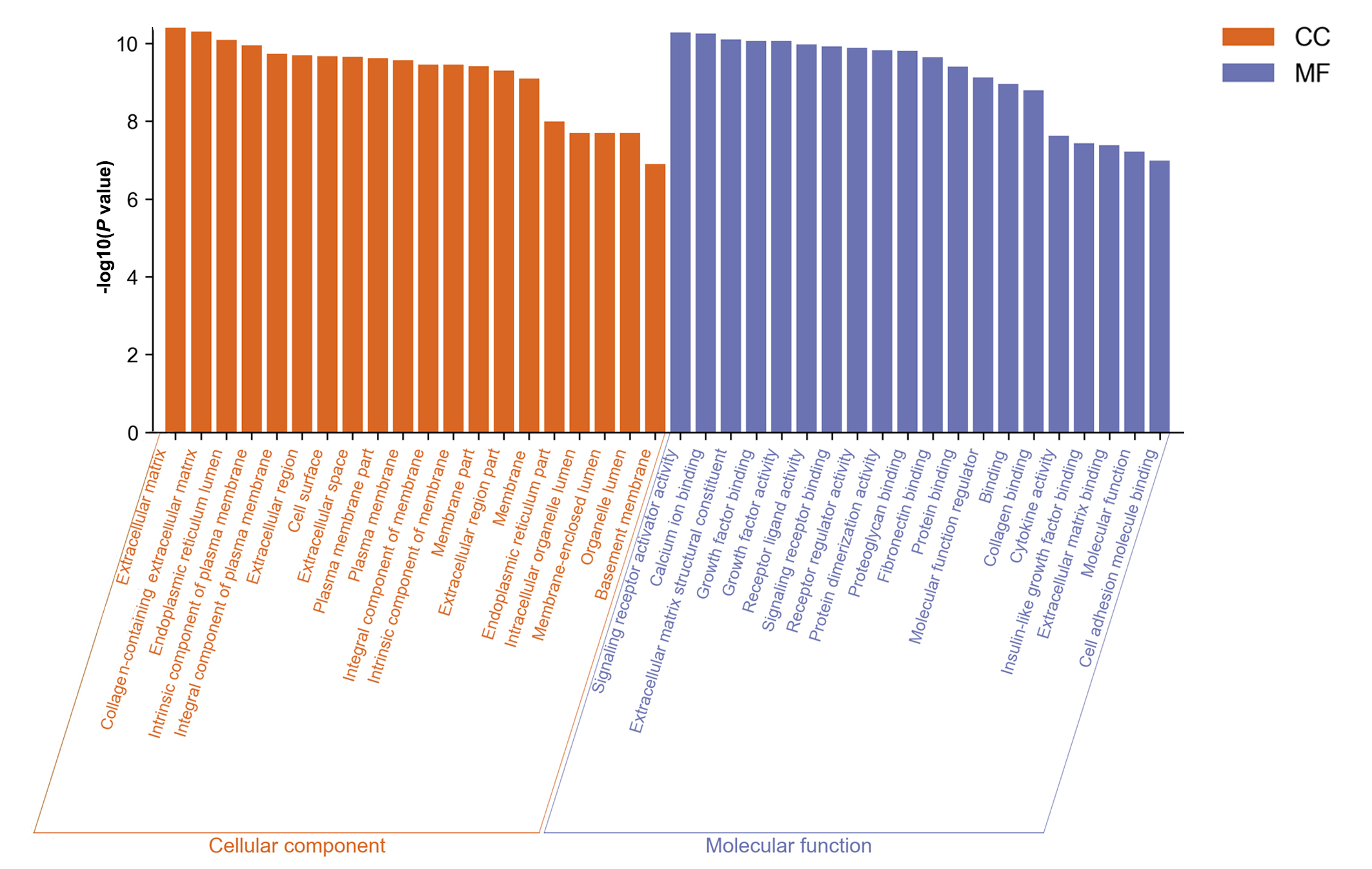

Supplement: Supplementary Figure 1 — Cellular component and molecular function of differentially expressed genes. The top 20 enriched cellular component terms and molecular function terms identified by GO (CC) and GO (MF) are shown based on differentially expressed genes. [file Image_1.jpg]

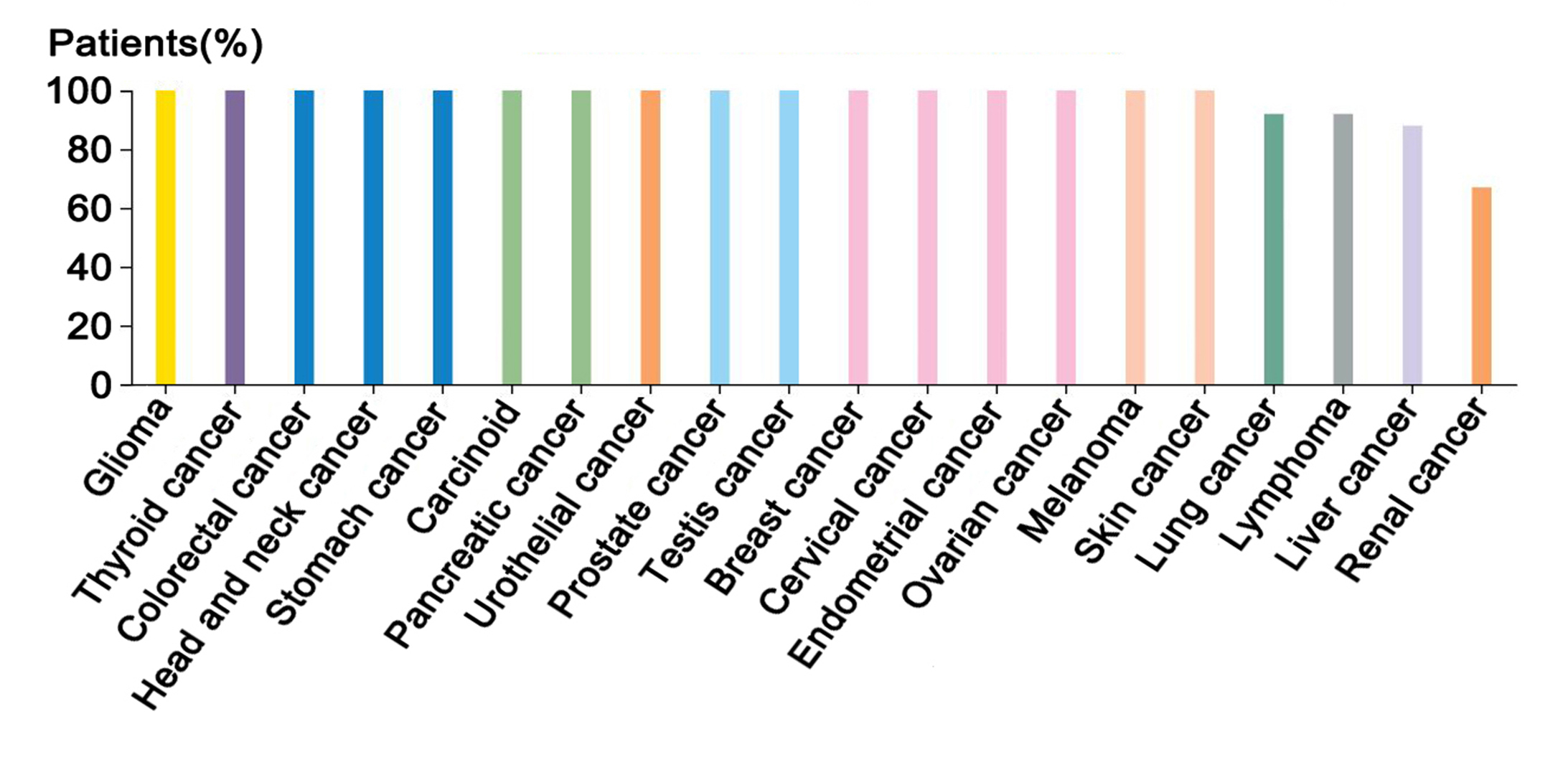

Supplement: Supplementary Figure 2 — Most cancer cells from the HPA showed strong nuclear immunoreactivity. [file Image_2.jpg]

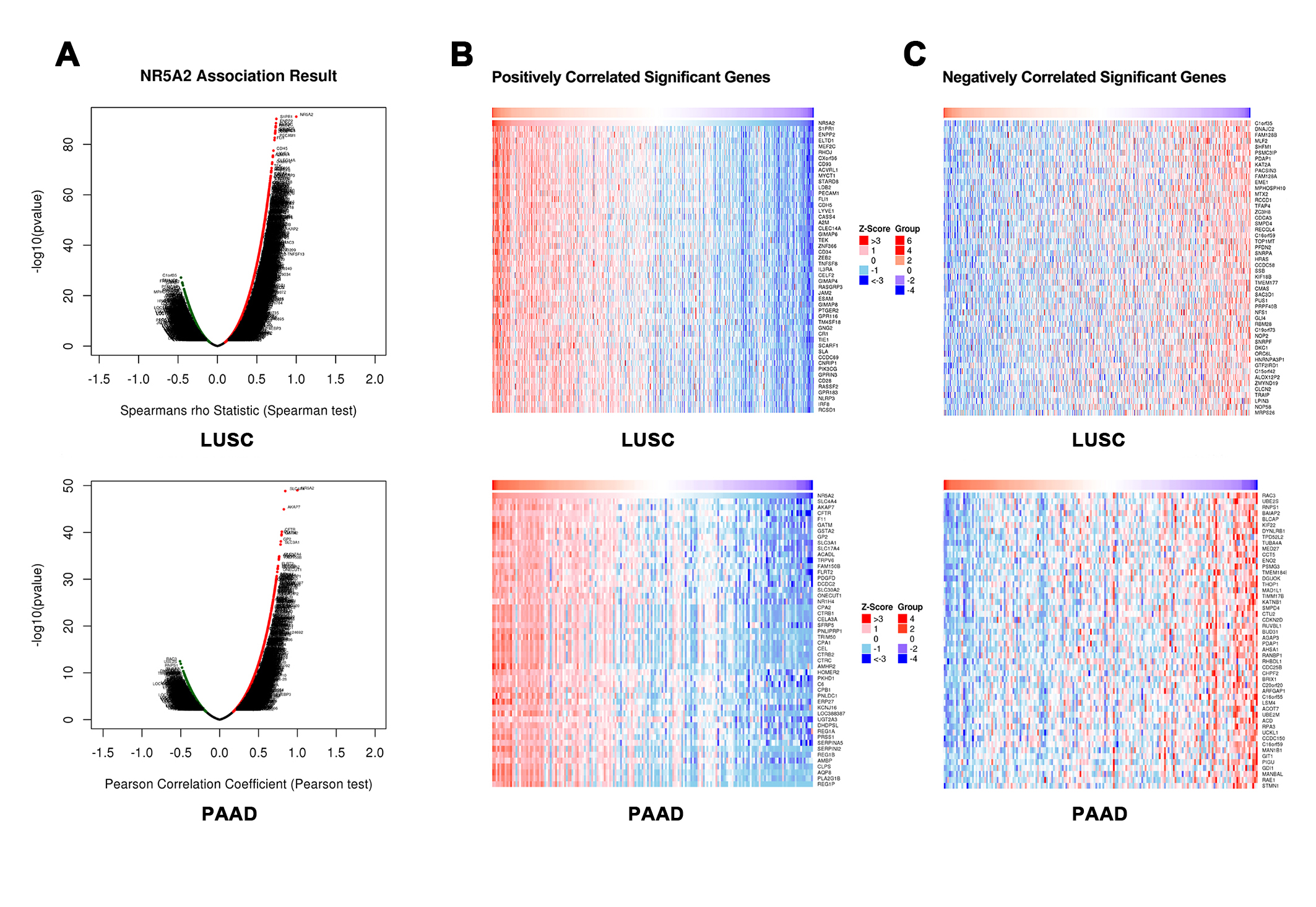

Supplement: Supplementary Figure 3 — Significant genes related to LRH1. (A) A Pearson test was used to analyze correlations between LRH1 and genes differentially expressed in LUSC (upper panel) and PAAD (lower panel). Heat maps of (B) and (C) show genes positively and negatively correlated with LRH1 in LUSC and PAAD, respectively (top 50). Red indicates positively correlated genes, and green indicates negatively correlated genes. [file Image_3.jpg]

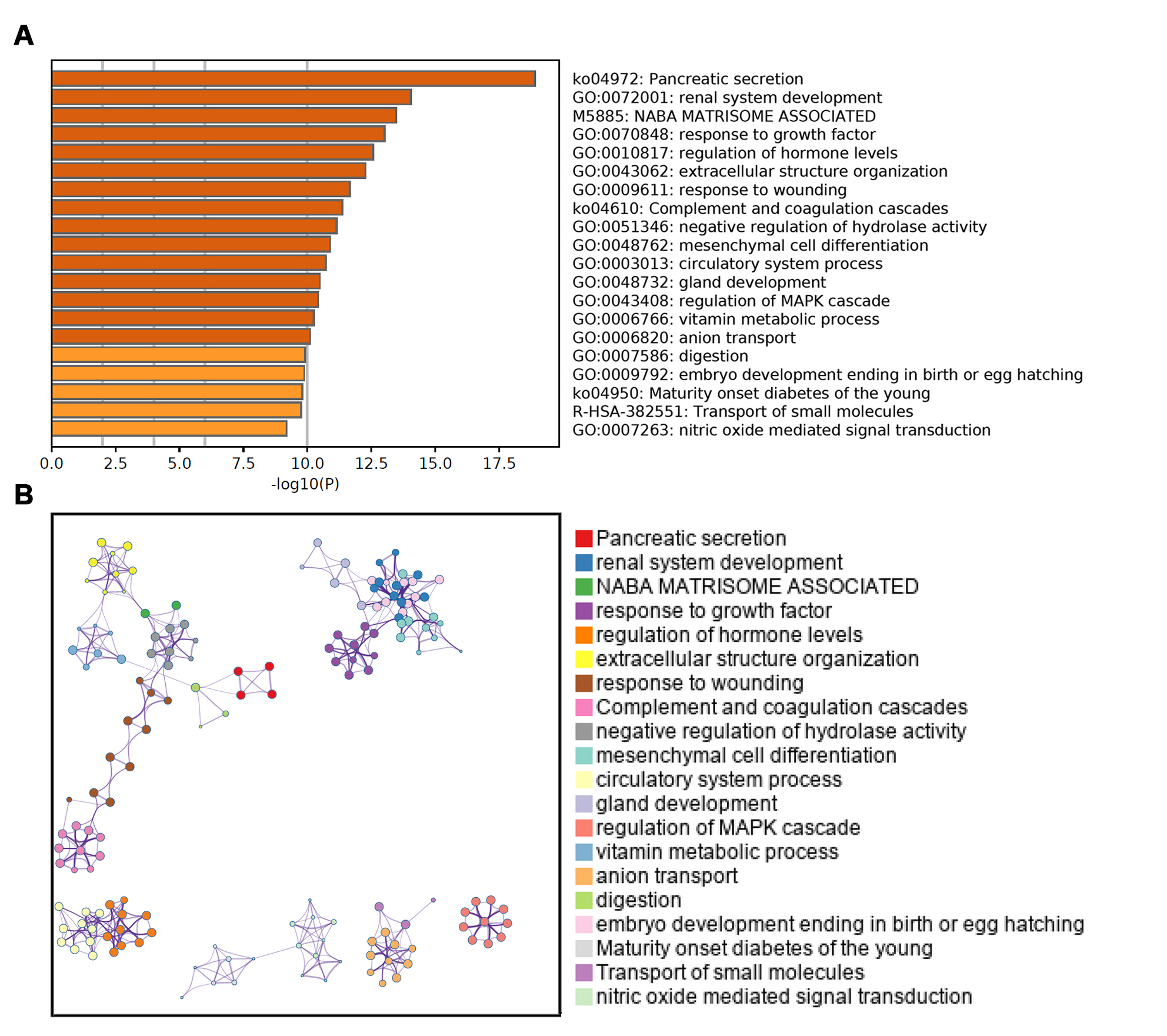

Supplement: Supplementary Figure 4 — Functional enrichment analysis of LRH1-related genes in pancreatic cancer. (A) A heatmap is shown of enriched terms regarding Gene Ontology across LRH1 and its related genes constructed by Metascape. (B) An interactive network shows the top 20 enriched terms colored by cluster ID. Each color represents one enrichment pathway. [file Image_4.jpg]

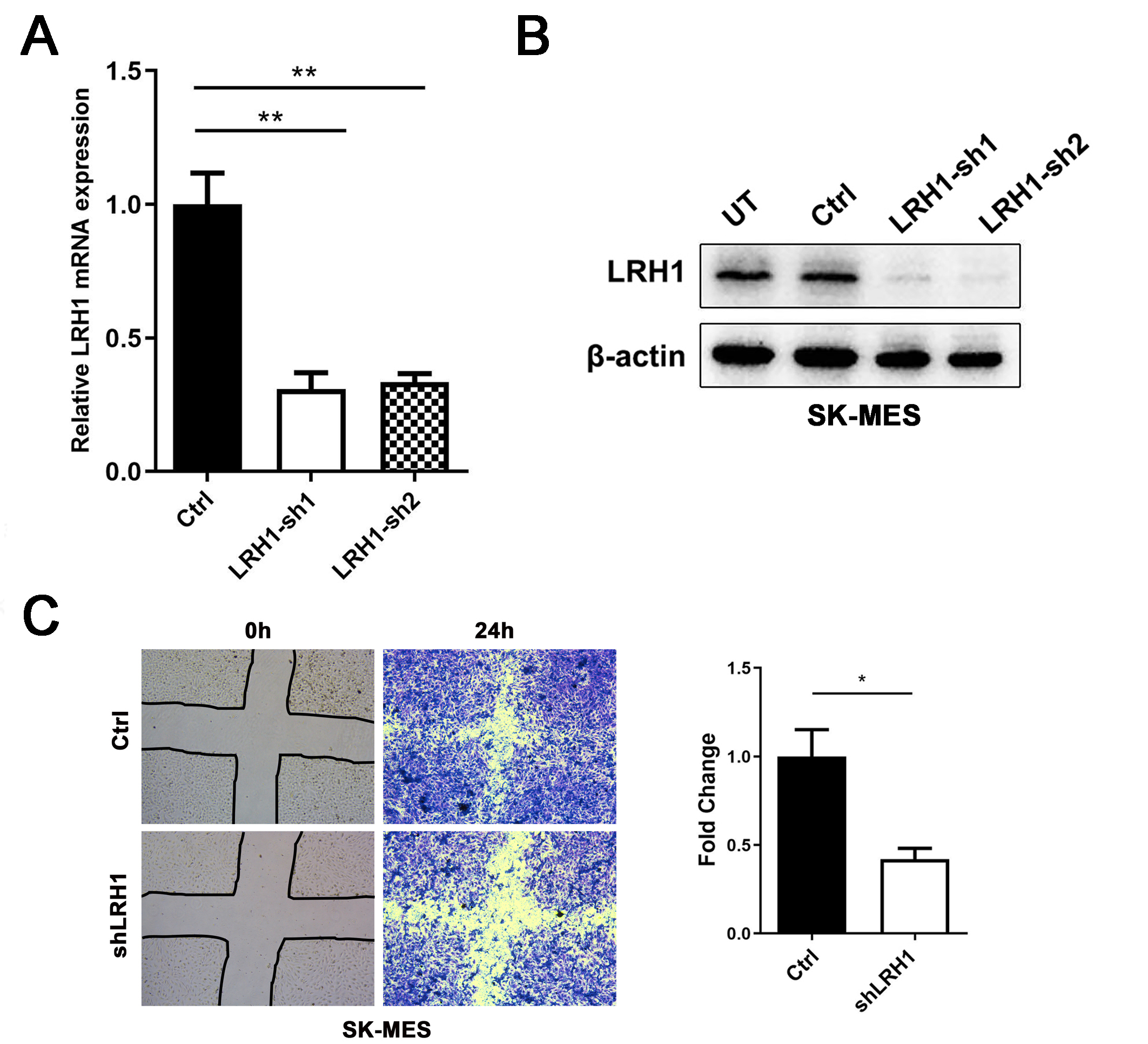

Supplement: Supplementary Figure 5 — LRH1 depletion suppresses the motility and migration of LUSC cells. (A) RT-PCR and (B) Western blot analysis results are shown of LRH1 in SK-MES cells untreated (UT) or transfected with control shRNA (Ctrl) or two shRNAs (LRH1-sh1 and LRH1-sh2) against LRH1. Mean ± SD, n = 3, *P < 0.05. (C) Wound healing assays indicate the motility and migration of LUSC cells in the shLRH1 group and control group. **P < 0.01 versus Ctrl, by 2-way ANOVA with Tukey’s t test. [file Image_5.jpg]

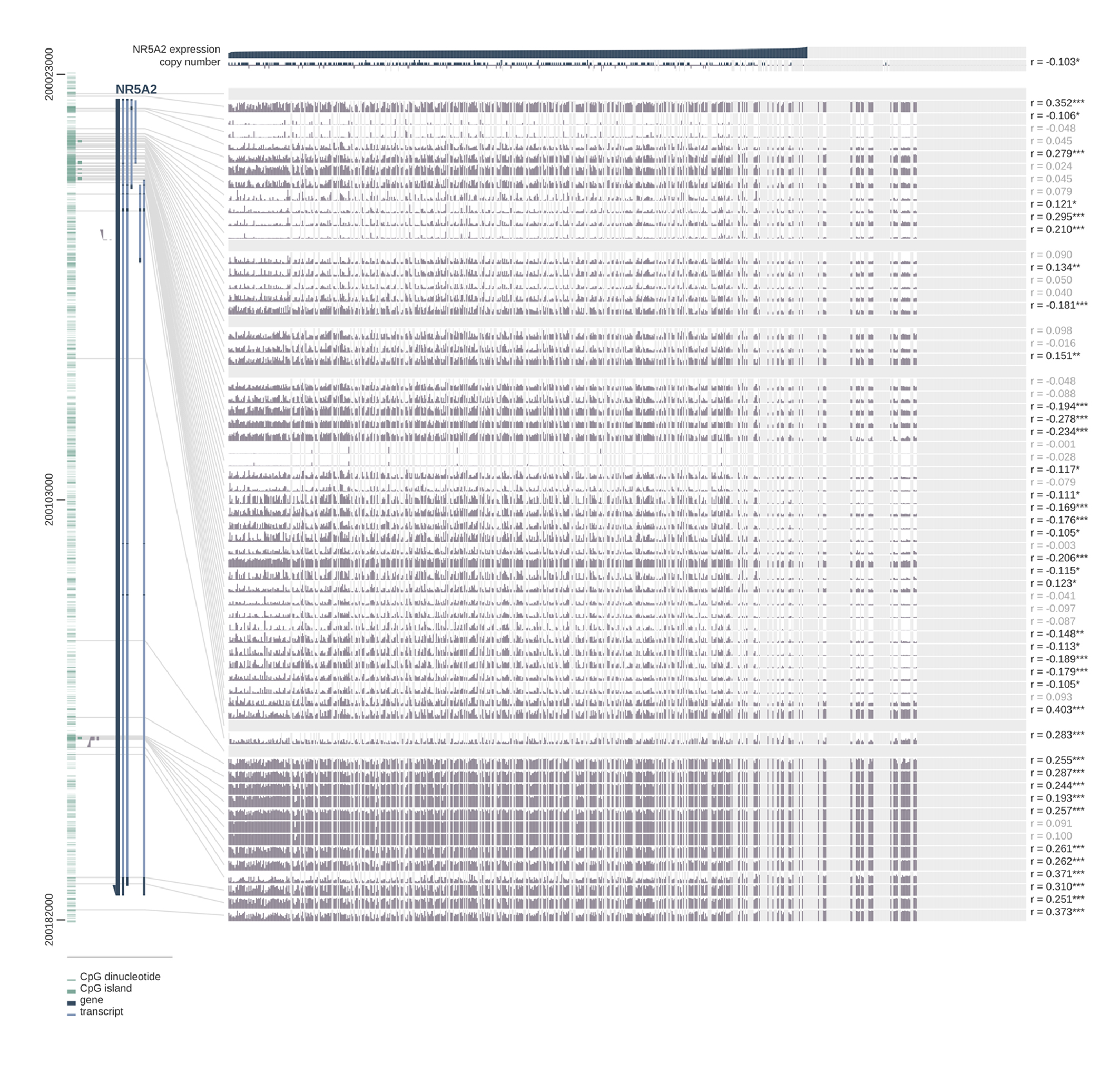

Supplement: Supplementary Figure 6 — The correlation of LRH1 expression and DNA methylation of the LRH1 promoter region including 758 samples in LUAD from TCGA datasets. The statistics [correlation coefficient (r) and P-value] on the right show the relationship between expression and DNA methylation of the promoter (*<0.05, **<0.01, and ***<0.001). [file Image_6.jpg]

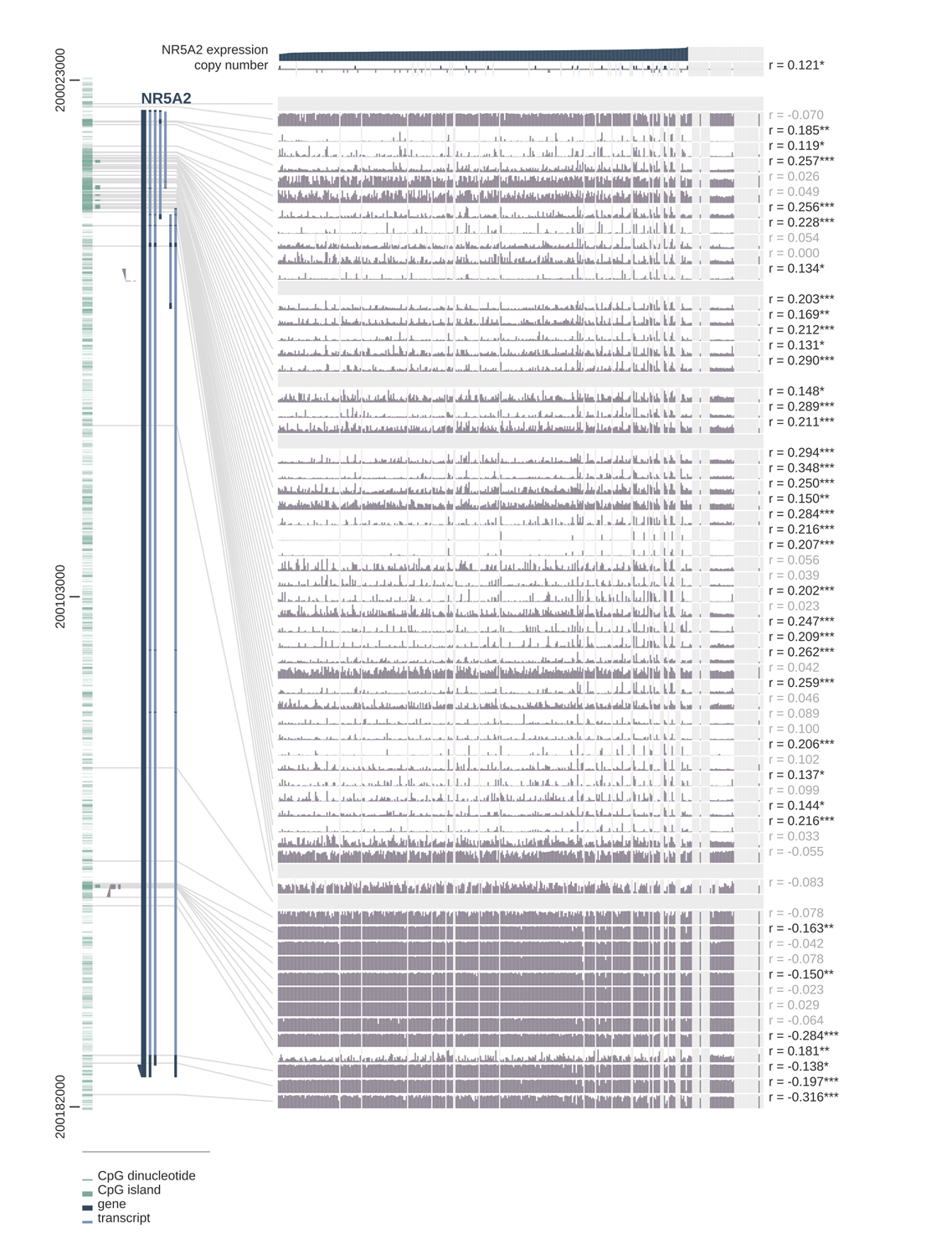

Supplement: Supplementary Figure 7 — The correlation of LRH1 expression and DNA methylation of the LRH1 promoter region including 380 samples in KIRP from TCGA datasets. The statistics [correlation coefficient (r) and P-value] on the right show the relationship between expression and DNA methylation of the promoter (*<0.05, **<0.01, and ***<0.001). [file Image_7.jpg]
