# Supplementary material for: LRH1 Acts as an Oncogenic Driver in Human Osteosarcoma and Pan-Cancer
Source: Front Cell Dev Biol. 2021 Mar 15;9:643522. doi: 10.3389/fcell.2021.643522 (PMC8005613; doi:10.3389/fcell.2021.643522)
Supplement: Supplementary Table 1 — The association between LRH1 expression and clinicopathological characteristics of OS patients. [file Table_1.docx]

| Table 1. Association between LRH1 expression and clinicopathological characteristics of OS patients. | | | | |
| --- | --- | --- | --- | --- |
|  |  | LRH1 expression | |  |
| Variables | No. of Patients | Low | High | *P*-value |
| Gender |  |  |  | 0.370 |
| Female | 14 | 6 | 8 |  |
| Male | 26 | 15 | 11 |  |
| Age |  |  |  | 0.698 |
| ＜18 years | 16 | 9 | 7 |  |
| ≥18 years | 24 | 12 | 12 |  |
| Location |  |  |  | 0.330† |
| Femur | 26 | 16 | 10 |  |
| Tibia | 5 | 2 | 3 |  |
| Other sites | 9 | 3 | 6 |  |
| Differentiation |  |  |  | 0.005* |
| Well/Moderate (G1/2) | 22 | 16 | 6 |  |
| Poor (G3) | 18 | 5 | 13 |  |
| T stage |  |  |  | 1† |
| T1 | 7 | 4 | 3 |  |
| T2 | 33 | 17 | 16 |  |
| TNM stage |  |  |  | 0.01*† |
| I | 22 | 16 | 6 |  |
| II | 17 | 5 | 12 |  |
| III/IV | 1 | 0 | 1 |  |

Abbreviations: OS = osteosarcoma, TNM stage = tumor, node, metastasis stage, No. = number of patients. *P* value: the difference of clinicopathological characteristics between the LRH1 high expression group and low expression group. **P* < 0.05 was considered statistically significant. † Fisher’s exact test.
